# Advanced glycation end product cross-link breaker attenuates diabetes-induced cardiac dysfunction by improving sarcoplasmic reticulum calcium handling

**DOI:** 10.3389/fphys.2012.00292

**Published:** 2012-07-19

**Authors:** Allyson L. Kranstuber, Carlos del Rio, Brandon J. Biesiadecki, Robert L. Hamlin, Joseph Ottobre, Sandor Gyorke, Véronique A. Lacombe

**Affiliations:** ^1^College of Pharmacy, The Ohio State UniversityColumbus, OH, USA; ^2^Department of Animal Sciences, The Ohio State University College of Food, Agriculture and Environmental SciencesColumbus, OH, USA; ^3^QTest LabsColumbus, OH, USA; ^4^The Dorothy M. Davis Heart and Lung Research Institute, The Ohio State UniversityColumbus, OH, USA; ^5^Department of Physiology and Cell Biology, The Ohio State University College of MedicineColumbus, OH, USA; ^6^College of Veterinary Medicine, The Ohio State UniversityColumbus, OH, USA; ^7^Department of Physiological Sciences, Oklahoma State UniversityStillwater, OK, USA

**Keywords:** cardiomyopathy, sarcoplasmic reticulum Ca^2+^-ATPase pump, diastolic function, type 1 diabetes, alagebrium chloride (ALT-711)

## Abstract

Diabetic heart disease is a distinct clinical entity that can progress to heart failure and sudden death. However, the mechanisms responsible for the alterations in excitation-contraction coupling leading to cardiac dysfunction during diabetes are not well known. Hyperglycemia, the landmark of diabetes, leads to the formation of advanced glycation end products (AGEs) on long-lived proteins, including sarcoplasmic reticulum (SR) Ca^2+^ regulatory proteins. However, their pathogenic role on SR Ca^2+^ handling in cardiac myocytes is unknown. Therefore, we investigated whether an AGE cross-link breaker could prevent the alterations in SR Ca^2+^ cycling that lead to *in vivo* cardiac dysfunction during diabetes. Streptozotocin-induced diabetic rats were treated with alagebrium chloride (ALT-711) for 8 weeks and compared to age-matched placebo-treated diabetic rats and healthy rats. Cardiac function was assessed by echocardiographic examination. Ventricular myocytes were isolated to assess SR Ca^2+^ cycling by confocal imaging and quantitative Western blots. Diabetes resulted in *in vivo* cardiac dysfunction and ALT-711 therapy partially alleviated diastolic dysfunction by decreasing isovolumetric relaxation time and myocardial performance index (MPI) (by 27 and 41% vs. untreated diabetic rats, respectively, *P* < 0.05). In cardiac myocytes, diabetes-induced prolongation of cytosolic Ca^2+^ transient clearance by 43% and decreased SR Ca^2+^ load by 25% (*P* < 0.05); these parameters were partially improved after ALT-711 therapy. SERCA2a and RyR2 protein expression was significantly decreased in the myocardium of untreated diabetic rats (by 64 and 36% vs. controls, respectively, *P* < 0.05), but preserved in the treated diabetic group compared to controls. Collectively, our results suggest that, in a model of type 1 diabetes, AGE accumulation primarily impairs SR Ca^2+^ reuptake in cardiac myocytes and that long-term treatment with an AGE cross-link breaker partially normalized SR Ca^2+^ handling and improved diabetic cardiomyopathy.

## Introduction

Diabetes has become an epidemic disease and it is estimated that by the year 2025, it will affect over 300 million people worldwide (Amos et al., 1997; Boudina and Abel, 2007). In the United States alone, about 8% of the population is affected by diabetes and approximately one million of those people suffer from insulin-dependent (type 1) diabetes. Type 1 diabetes is characterized by sustained hyperglycemia resulting from the loss of insulin-producing pancreatic beta cells. This loss in insulin production results in dysfunctional glucose uptake in insulin-sensitive tissues (e.g., striated muscle) and causes multiple-organ complications. Of importance, diabetes is also a common cause of cardiovascular diseases. Within the past 30 years, diabetic cardiomyopathy has been identified as its own clinical unit, independent of coronary artery disease and atherosclerosis (Fang et al., 2004; Poornima et al., 2006). Ventricular diastolic dysfunction is the first stage of diabetic cardiomyopathy and has been reported in about 50% of asymptomatic patients (Fang et al., 2004; Lacombe et al., 2007). Because intracellular calcium (Ca^2+^) homeostasis is crucial for excitation-contraction coupling, chronic diabetes mellitus has been associated with impaired cardiac contractility and relaxation of the myocardium due to altered Ca^2+^ homeostasis (Lagadic-Gossmann et al., 1996; Pierce and Russell, 1997; Netticadan et al., 2001; Zhong et al., 2001; Choi et al., 2002; Fang et al., 2004; Lacombe et al., 2007). However, the exact mechanisms for this impaired Ca^2+^ homeostasis and the specific therapeutic strategies for this patient population remain elusive.

The sarcoplasmic reticulum (SR) functions as the main regulator of intracellular Ca^2+^ and is a major determinant of cardiac contraction and relaxation (Bers, 2002). Ca^2+^ entry through the L-type Ca^2+^ channel activates Ca^2+^ release from the SR. The SR Ca^2+^ release channels, the ryanodine receptors (RyRs), release the majority of free Ca^2+^ necessary for contraction, and SR Ca^2+^ ATPase (SERCA2a) pumps sequester the majority of Ca^2+^ during relaxation of cardiac myocytes. Several groups (including ours) have reported decreased expression of these SR Ca^2+^ regulatory proteins in type 1 diabetic rats with cardiac dysfunction (Poornima et al., 2006; Lacombe et al., 2007; Ratnadeep et al., 2009). Furthermore, impaired excitation-contraction coupling in diabetic myocytes has been characterized by slower Ca^2+^ transient decays and cytosolic Ca^2+^ overload during the diastolic phase (Pierce and Russell, 1997; Choi et al., 2002; Lacombe et al., 2007). However, the mechanisms by which SR Ca^2+^ cycling is impaired during diabetic cardiomyopathy have not been fully elucidated.

Chronic hyperglycemia, the hallmark of diabetes, accelerates the reaction between glucose and proteins and leads to the formation of advanced glycation end products (AGEs). These AGEs form irreversible cross-links throughout the lifetime of many large proteins (such as collagen and hemoglobin), covalently modifying their structure and function (Cooper, 2004). Therefore, AGEs induce myocardial fibrosis and stiffness leading to severe cardiac dysfunction (Norton et al., 1996; Asif et al., 2000; Vaitkevicius et al., 2001; Aronson, 2003; Candido et al., 2003; Bakris et al., 2004; Cooper, 2004; Hartog et al., 2007; Ma et al., 2009). In addition, Bidasee et al. (2003, 2004) have demonstrated the presence of cross-linked AGEs on long-lived intracellular cardiac SR proteins such as the SERCA2a pump and RyR2 after a few weeks of diabetes. Therefore, one could hypothesize that the post-translational modifications of the SR proteins by AGEs could lead to an alteration in Ca^2+^ homeostasis. However, the *functional significance* of AGEs on SR Ca^2+^ regulatory proteins in cardiac myocytes and thus on excitation-contraction coupling has not been determined. Our hypothesis was that treatment with an antiglycation therapeutic agent, dimethyl-3-phenacylthiazolium chloride (alagebrium chloride or ALT-711), which chemically breaks AGE cross-links, will normalize SR Ca^2+^ reuptake in cardiac myocytes and therefore improve diastolic function in type 1 diabetes.

## Materials and methods

### Animal model

Eight-week-old male Wistar rats were randomly divided into 3 groups (*n* = 11/group): untreated age-matched control group (CON); untreated diabetic group (DX); and ALT-711 (Shanghai Inc., China) treated diabetic group (DX-ALT). Diabetes was induced at 10 weeks of age in DX and DX-ALT groups by a single injection of streptozotocin (STZ, 50 mg/kg IP diluted in 1 mL citrate buffer). The control group received similar volume of vehicle. One diabetic group received dimethyl-3-phenacylthiazolium chloride (ALT-711, 10 mg/kg per day in the drinking water) for 8 weeks. This therapeutic dose has been previously shown to significantly reduce cardiac AGE level in STZ-induced diabetes (Candido et al., 2003). The volume of ALT-711 delivered in the drinking water was calculated based on the individual water consumption, which was measured every other day. To confirm the status of diabetes, venous blood samples were drawn from the tail vein for measurement of blood glucose concentration using a glucometer (BD Logic) at baseline and then weekly after STZ injection for 8 weeks. Animals were weighed once a week, as a means to monitor their clinical condition. This animal protocol was approved by the Ohio State University Institutional Animal Care and Use Committee.

### Echocardiography

Transthoracic echocardiographic examination was performed to assess systolic and diastolic function at baseline and 8 weeks after the induction of diabetes. Two-dimensional, M-mode, and pulsed-wave Doppler imaging were obtained in rats lightly anesthetized with isoflurane (minimal effective concentration), and placed on a heating table to maintain normothermia. Examinations were done using a high-resolution high-frequency digital imaging system with a 21 MHz linear-array transducer and simultaneous ECG recording (Vevo 2100, VisualSonics, Toronto, Canada), following standard techniques as previously described (Dirksen et al., 2007; Lacombe et al., 2007, 2010; Ware et al., 2011). Standard parasternal long- and short-axis views (6–8/rat) were obtained during each echocardiographic examination. Ventricular structure and function were assessed by two-dimensional cine loops of a long-axis view (with frame rates of at least 200 frames/s) and of a short-axis view at mid-level of the papillary muscles, as well as M-mode loops of the short-axis view. Thicknesses of the interventricular septum and of the left ventricular posterior wall, and left ventricular internal diameter (LVID) were measured in systole and diastole from the short-axis view according to standard procedures. Left ventricular (LV) ejection fraction (EF), a surrogate of systolic function, was calculated, as follows: EF = (LVID end-diastolic – LVID end-systolic/LVID end-diastolic) × 100%. The apical four-chamber view was used for color flow guided, pulsed-wave Doppler imaging of transmitral flow and LV outflow. The myocardial performance index (MPI or TEI index) was obtained from the sum of the LV isovolumic relaxation time and isovolumic contraction time divided by the aortic ejection time, parameters which were measured from the pulsed-wave Doppler imaging of transmitral flow and LV outflow. Echocardiographic image measurements were performed offline. All image acquisitions and offline measurements were conducted by the same investigator (AK). Average values were obtained from the measurement of three cardiac cycles from one cine loop.

### Masson trichrome staining

LV fibrosis was measured at 8 weeks after the induction of diabetes by the Ohio State University's Core Pathology laboratory. LV cross sections were washed with PBS, fixed using OCT (optimal cutting temperature) compound, frozen in dry ice and stained with Masson Trichrome staining.

### Ventricular myocyte isolation

Following echocardiographic measurements, animals were euthanized by pentobarbital sodium. The heart was removed and perfused in a retrograded manner, using a Langendorff apparatus with tyrode buffer (37°C, pH = 7.35 and oxygenated with 95% O_2_ and 5% CO_2_), which contained (in mM): NaCl (135), KCl (5.4), MgCl_2_ (1), NaH_2_PO_4_ (0.33), Hepes (10), glucose (10), and CaCl_2_ (1). This initial perfusion was followed by a perfusion with tyrode buffer without any CaCl_2_. Subsequently, collagenase (type II, Worthington Biochemical, 1 mg/ml) was added to the calcium free tyrode buffer and recirculated for the rest of the perfusion period. When the heart was soft, the ventricles were minced and the cells were subsequently washed in tyrode solution containing CaCl_2_ (1). Only rod-shaped cells with sharp margins and clear striations were included in the study. All recordings were made within 5 h of isolation (Dirksen et al., 2007; Lacombe et al., 2007, 2010).

### Measurement of Ca^2+^ transient and SR Ca^2+^ load

Ca^2+^ transient was measured in fluo-3-loaded cardiac myocytes with confocal Ca^2+^ imaging as previously described; for measurements of Ca^2+^ transients and transient decay, mean area under the curve was calculated (Kubalova et al., 2005; Dirksen et al., 2007; Lacombe et al., 2007, 2010). Rapid applications of caffeine (10 mM) were used to measure SR Ca^2+^ content by measuring the peak amplitude of the caffeine-induced Ca^2+^ transients. Intracellular Ca^2+^ imaging was performed using a Laser Scanning Confocal System (Olympus Fluoview 1000 confocal microscope interfaced to an IX-70 inverted microscope and equipped with an 60 × 1.4 NA oil objective). Fluo-3 was excited by the 488- nm beam of an argon-ion laser, and the fluorescence was acquired at wavelengths > 515 nm in the line scan mode, at a rate of 2 or 6 ms per scan. The magnitude of fluorescent signals was quantified in terms of F/F0, where F0 is baseline fluorescence (Kubalova et al., 2005; Dirksen et al., 2007; Lacombe et al., 2007, 2010).

### Measurements of SR Ca^2+^ regulatory proteins

LV myocardium was collected 8 weeks after the induction of diabetes. Crude membrane homogenates were prepared for Western blot analysis, as previously described (Meurs et al., 2006; Lacombe et al., 2007; Ware et al., 2011). Proteins were subjected to sodium dodecyl sulphate-polyacrylamide gel electrophoresis (SDS-PAGE) electrophoresis, electrophoretically transferred to PVDF membranes using a trans-blot cell (Bio-Rad Laboratories, Hercules, CA, USA; Meurs et al., 2006; Lacombe et al., 2007; Ware et al., 2011). Samples from the 3 groups were loaded on the same gel to ensure equal blotting conditions for each group. Membrane proteins were incubated with mouse RyR2 or SERCA2a antibodies (1:3000 and 1:1000 dilution, respectively, Affinity Bioreagents), and subsequently with the appropriate secondary antibodies conjugated to horseradish peroxidase (1:50,000 dilution, Jackson ImmunoResearch Laboratories; 1:5000 dilution, Sigma Aldrich, respectively). Quantitative determination of protein was performed by autoradiography after revealing the antibody-bound protein by enhanced chemiluminescence reaction. The data were normalized to actin or calsequestrin, previously quantified by reprobing each membrane with calsequestrin polyclonal IgG (Calbiochem) or Actin monoclonal IgG (Sigma Aldrich), respectively.

### Statistical analysis

A Two-Way ANOVA (treatment and time factors) for the *in vivo* measurements, and a one-way analysis of variance (treatment factor) for the *in vitro* measurements were performed, as appropriate. Data were reported as means ± SE. Statistical significance was defined as *P* < 0.05.

## Results

**Figure 1:**
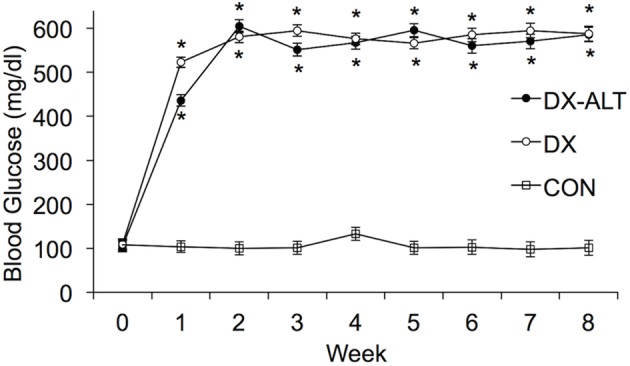
**Blood glucose was significantly increased in DX and DX-ALT groups compared to age-matched control group.** Mean ± SE of blood glucose concentration in control (CON), untreated diabetic (DX), treated diabetic (DX-ALT) rats at baseline (time 0) and up to 8 weeks after the induction of diabetes. *n* = 10 − 11/group. ^*^P < 0.05 when comparing values from age-matched controls.

**Figure 2:**
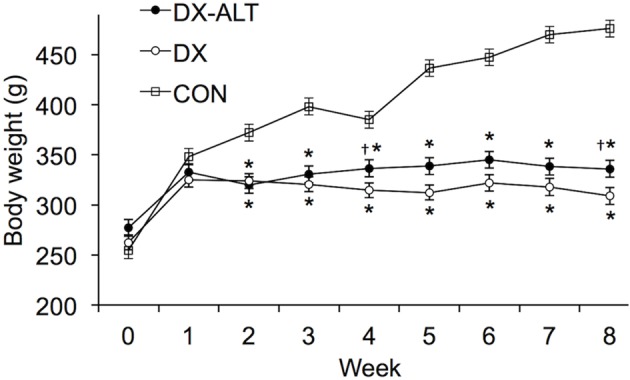
**Body weight was significantly decreased in DX and DX-ALT groups compared to age-matched control group.** Mean ± SE of body weight in control (CON), untreated diabetic (DX), treated diabetic (DX-ALT) rats at baseline (time 0) and 8 weeks after the induction of diabetes. *n* = 10 − 11/group. ^*^P < 0.05 when comparing values from age-matched controls. ^†^*P* < 0.1 when comparing values from DX group.

As expected, the STZ-treated rats exhibited hyperglycemia within 72 h post injection, which persisted during the 8-week experimental period (*P* < 0.05, Figure 1). Treatment with an AGE cross-link breaker, ALT-711, for 8 weeks did not significantly alter blood glucose concentration compared with untreated diabetic rats. In addition, the diabetic rats had a significantly lower body weight when compared to the control group and there was a tendency (*P* < 0.1) for the treated diabetic rats to have a higher body weight than the untreated diabetic rats at 4 and 8 weeks after the induction of diabetes (Figure 2).

### *In vivo* ventricular function

**Figure 3:**
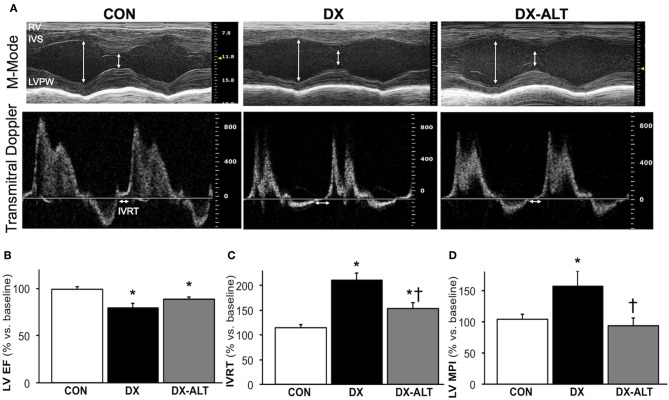
**ALT-711 therapy partially alleviated diabetic cardiomyopathy by primarily improving Doppler-derived parameters of diastolic function. (A)** A representative paired M-mode echocardiograms (*top panel*) and transmitral Doppler flow (*bottom panel*) at 8 weeks of treatment in control (Con), untreated diabetic (DX), and treated diabetic (DX-ALT) groups. IVS, interventricular septum; LVPW, left ventricular posterior wall; RV, right ventricle; IVRT: isovolumic relaxation time. **(B)** Mean ± SE of percentage change after 8 weeks of treatment compared to baseline value for ejection fraction (EF) of the left ventricle (LV) in control (Con), untreated diabetic (DX), and treated diabetic (DX-ALT) groups. **(C)** Mean ± SE of percentage change at 8 weeks compared to baseline value for isovolumic relaxation time (IVRT) of the left ventricle in control (Con), untreated diabetic (DX), and treated diabetic (DX-ALT) groups. **(D)** Mean ± SE of percentage change at 8 weeks compared to baseline value for myocardial performance index (MPI) in control (Con), untreated diabetic (DX), and treated diabetic (DX-ALT) groups. *n* = 9 − 11/group, ^*^P < 0.05 when comparing values from age-matched controls. ^†^*P* < 0.05 when comparing values from DX group.

We then evaluated the effect of diabetes and ALT-711 therapy on systolic and diastolic function by echocardiographic examination in (treated and untreated) diabetic and control groups. EF, a surrogate of systolic function, was mildly decreased at 8 weeks after the induction of diabetes compared to baseline values (Table 1). In addition, the isovolumic relaxation time and the MPI, two parameters of LV relaxation, were significantly increased in untreated diabetic rats compared to the age-matched control groups (Table 2). ALT-711 therapy did not significantly alter EF of the diabetic myocardium but blunted the increase in isovolumic relaxation time and the MPI in diabetic animals, suggesting that ALT-711 therapy partially prevented cardiac dysfunction by primarily improving diastolic dysfunction (Figure 3 and Table 2).

**Table 1:** **Parameters derived from M-mode echocardiography of LV for age-matched control (CON), untreated diabetic (DX), treated diabetic (DX-ALT) groups at baseline and at 8 weeks after the induction of diabetes**.

	**CON**		**DX**		**DX-ALT**	
	**Baseline**	**8 weeks**	**Baseline**	**8 weeks**	**Baseline**	**8 weeks**
EF (%)	85.7 ± 2.6	84.4 ± 1.8	86.11 ± 2.02	67.8 ± 2.54^*^^#^	83.7 ± 2.08	73.8 ± 0.14^*^^#^
FS (%)	57.7 ± 3.11	55.7 ± 2.22	57.7 ± 2.50	39.6 ± 2.07^*^^#^	54.4 ± 2.42	44.5 ± 1.71^*^^#^
IVS;d (cm)	1.85 ± 0.07	2.11 ± 0.07	2.05 ± 0.07	1.85 ± 0.10	2.02 ± 0.10	1.84 ± 0.09
IVS;s (cm)	3.42 ± 0.07	3.76 ± 0.14^#^	3.64 ± 0.11	2.94 ± 0.09^*^^#^	3.54 ± 0.10	3.15 ± 0.09^*^^#^
LVID;d (cm)	6.46 ± 0.22	7.39 ± 0.13^#^	6.47 ± 0.16	7.74 ± 0.17^#^	6.59 ± 0.26	7.75 ± 0.2 ^#^
LVID;s (cm)	2.79 ± 0.29	3.29 ± 0.21^#^	2.74 ± 0.19	4.69 ± 0.22^*^^#^	3.00 ± 0.19	4.32 ± 0.20^*^^#^
LVPW;d (cm)	1.64 ± 0.07	1.84 ± 0.09	1.70 ± 0.08	1.63 ± 0.10	1.63 ± 0.08	1.60 ± 0.08
LVPW;s (cm)	3.17 ± 0.12	3.43 ± 0.1^#^	3.11 ± 0.12	2.57 ± 0.08^*^^#^	2.95 ± 0.12	2.67 ± 0.14^*^
LV Mass;c (mg)	610.1 ± 42.5	891.3 ± 36.2^#^	676.8 ± 35.8	812.9 ± 70.4^#^	663.2 ± 35.3	792.6 ± 41.2
LV Vol;d (ul)	216.5 ± 16.5	289.6 ± 11.3^#^	215.9 ± 11.45	322.1 ± 15.9^#^	226.6 ± 19.6	323.2 ± 18.4^#^
HR (b/min)	329.6 ± 9.21	318.24 ± 8.64	344.7 ± 2.00	243.8 ± 7.95^*^^#^	335.4 ± 10.5	268.3 ± 6.02^*^^#^

Data are mean ± SE for *n* = *9* – 11/group. IVS;s: interventricular septal dimension-systole, IVS;d: interventricular septal dimension-diastole, LVPW;s: left ventricular posterior wall dimension-systole, LVPW;d: left ventricular posterior wall dimension-diastole, LVID;s: left ventricular internal diameter-systole, LVID;d: left ventricular internal diameter-diastole; LV Mass;c: left ventricular mass corrected, EF: ejection fraction, FS: fractional shortening, HR: heart rate.

* P < 0.05 when comparing values from age-matched controls for same time point.

# P < 0.05 when comparing values from baseline.

**Table 2:** **Doppler-derived parameters of diastolic function for age-matched control (CON), untreated diabetic (DX), treated diabetic (DX-ALT) groups at baseline and at 8 weeks after the induction of diabetes**.

	**CON**		**DX**		**DX-ALT**	
	**Baseline**	**8 weeks**	**Baseline**	**8 weeks**	**Baseline**	**8 weeks**
MV E/A	1.52 ± 0.06	1.52 ± 0.06	1.62 ± 0.092	1.64 ± 0.080	1.59 ± 0.11	1.82 ± 0.15
IVRT	22.4 ± 0.83	25.4 ± 1.07	23.0 ± 0.95	44.98 ± 1.88^*^^#^	24.8 ± 1.14	37.3 ± 2.07^*^^†^
MV Dec Acc (mm/s^2^)	38647.0 ± 4166.0	34047.0 ± 4493.3	39465.7 ± 3418.6	39593.8 ± 8829.9	40454.0 ± 4650.1	26790.0 ± 2305.4
LV MPI	0.54 ± 0.04	0.57 ± 0.05	0.46 ± 0.03	0.76 ± 0.08^*^^#^	0.61 ± 0.04	0.56 ± 0.08^†^

Data are mean ± SE for n = 9 – 11/group. MV E, maximum velocity of the E wave; MV A, maximum velocity of the A wave; MV E/A, ratio of E and A wave; IVRT, isovolumic relaxation time; MV Dec Acc, maximum velocity deceleration acceleration; LV MPI, left ventricle myocardial performance index.

* P < 0.05 when comparing values from age-matched controls for same time point.

# P < 0.05 when comparing values from baseline.

† P < 0.05 when comparing DX values vs. DX-ALT.

**Figure 4:**
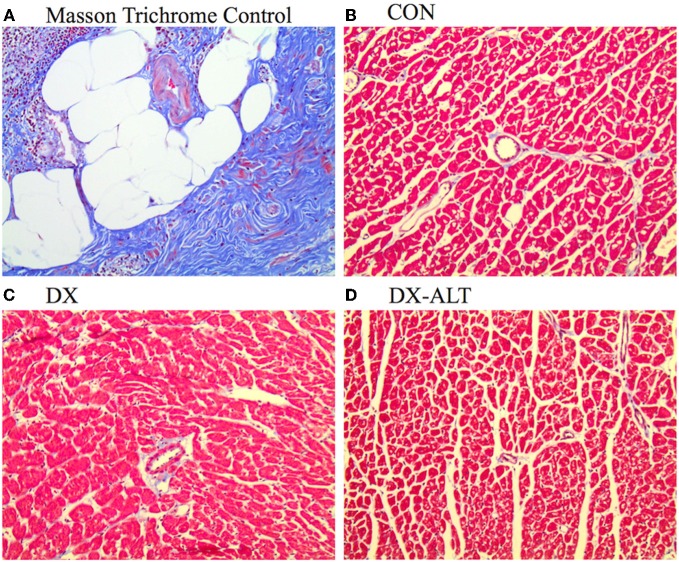
**Lack of fibrosis in ventricle of (untreated and treated) diabetic rats. (A)** Masson Trichrome staining showing fibrosis (Fibrosis Control experiment). **(B–D)** Representative Trichrome staining (×20 objective) demonstrating the lack of ventricle fibrosis in age-matched control (Con), untreated diabetic (DX), and treated diabetic (DX-ALT) groups.

Because diastolic dysfunction may be due to reduced rate of sequestration of Ca^2+^ into the SR or to histological changes in myocardium rendering it less compliant, hearts were stained with Masson's trichrome to quantify fibrosis. Masson trichrome staining did not reveal significant fibrosis of the left ventricle in (treated and untreated) diabetic rats compared with the control group, suggesting primarily an impaired ventricular relaxation rather than increased myocardial stiffness in our diabetic model (Figure 4).

### Measurement of Ca^2+^ transient and SR Ca^2+^ load

**Figure 5:**
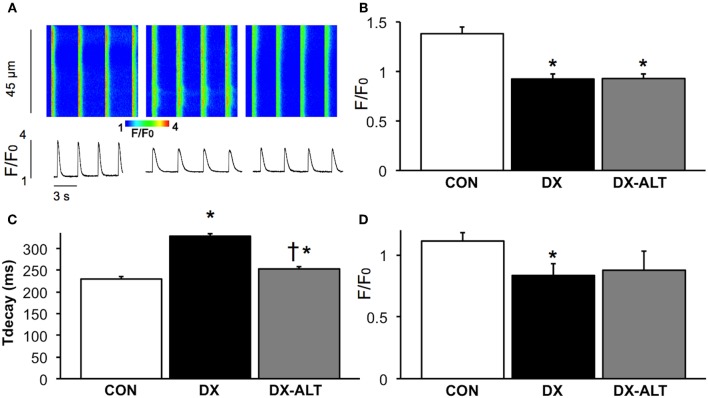
**ALT-711 therapy ablated the prolongation of Ca^2+^ transient decay in diabetic cardiac myocytes. (A)** Representative confocal line scan images of Ca^2+^ transient along with their spatial averages in myocytes from age-matched control (CON, left), untreated diabetic (DX, middle), and treated diabetic (DX-ALT, right) rats. F0, diastolic fluorescence. **(B)** Mean ± SE of Ca^2+^ transient amplitude (F/F0) for CON, DX, and DX-ALT rats, *n* = 43 − 44/group. **(C)** Mean ± SE of the time constant (τ) of Ca^2+^ transient decay in CON, DX, and DX-ALT rats. *n* = 40 ± 4/group. τ decay is significantly increased in DX compared to CON (*P* < 0.05). Please note a significant decrease in DX-ALT compared to DX, showing an improvement in calcium reuptake time after 8 weeks of ALT-711 treatment. **(D)** Caffeine-induced Ca^2+^ transient amplitudes (mean ± SE) were reduced in myocytes from diabetic compared with control and treated diabetic rats. *n* = 4 − 5/group, ^*^P < 0.05 when comparing values from age-matched control myocytes. ^†^*P* < 0.05 when comparing values from DX group.

Because AGEs accumulate on SR Ca^2+^ regulatory proteins and could alter their function leading to diabetic cardiomyopathy, we further determined if treatment with an AGE cross-link breaker would improve SR Ca^2+^ handling during diabetic cardiomyopathy by measuring Ca^2+^ transient in fluo-3-loaded cardiac myocytes with confocal Ca^2+^ imaging. There was a significant (*P* < 0.001) decrease in Ca^2+^ transient amplitude in isolated cardiac myocytes of (untreated and treated) diabetic rats when compared with controls (Figure 5). In addition, the Ca^2+^ transient decay during the diastolic phase was significantly prolonged in diabetic compared with control myocytes (Figure 5). ALT-711 therapy resulted in shortening of the Ca^2+^ transient decay, suggesting an improvement in SR Ca^2+^ reuptake in treated diabetic myocytes. In addition, SR Ca^2+^ load, measured by caffeine-evoked Ca^2+^ transient amplitudes, was significantly decreased in myocytes from untreated diabetic, but not from treated diabetic rats, compared with control groups (Figure 5), suggesting that ALT-711 therapy partially attenuated SR Ca^2+^ content depletion in diabetic myocytes.

### Measurement of SR Ca^2+^ regulatory proteins

**Figure 6:**
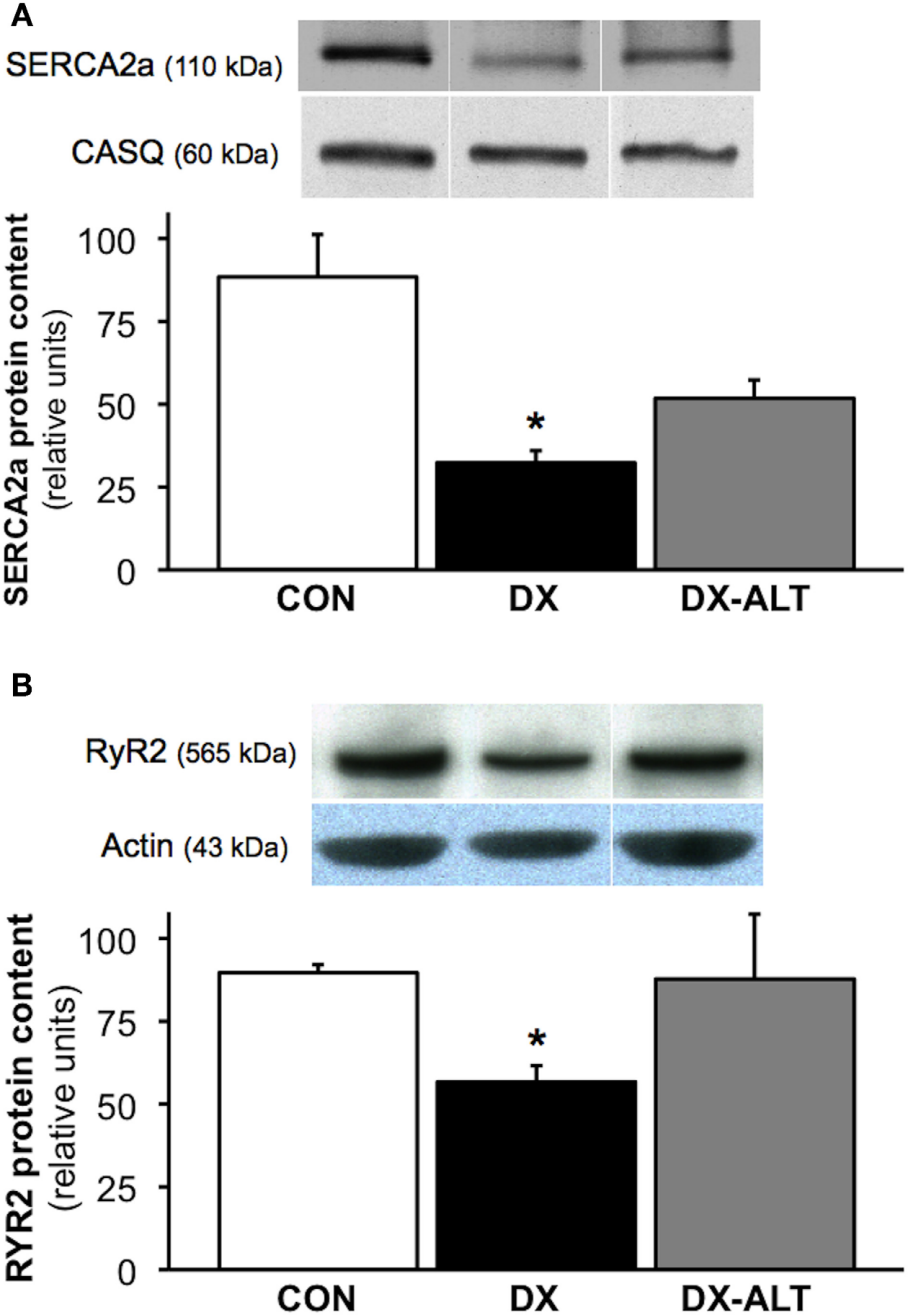
**ALT-711 therapy partially attenuated the decreased expression of SR Ca^2+^ regulatory proteins in the diabetic myocardium. (A)** *Top panel:* representative immunoblot of sarco(endo)plasmic reticulum Ca^2+^-ATPase (SERCA2a) and calsequestrin (loading control) expression in the myocardium of control (CON), untreated diabetic (DX), treated diabetic (DX-ALT) groups; samples were from the same membrane, which was reprobed for calsequestrin (the loading control). Within the same gel, the reassembly of noncontiguous lanes has been demarcated by white spaces. *Bottom panel:* normalized optical density (OD; relative to calsequestrin) of SERCA2a protein content was significantly decreased in DX, but not in DX-ALT, hearts compared to control group. Data are mean ± SE for *n* =4 − 5/group. **(B)** *Top panel:* representative immunoblot of Ryanodine Receptor (RyR2) protein expression in the myocardium of control (CON), untreated diabetic (DX), treated diabetic (DX-ALT) hearts (samples were from the same membrane, which was reprobed for actin, the loading control. Within the same gel, the reassembly of noncontiguous lanes has been demarcated by white spaces). *Bottom panel:* normalized optical density (relative to actin, loading control) of RyR2 protein was significantly decreased in DX, but unchanged in DX-ALT, hearts compared to control group. Data are mean ± SE for *n* = 5/group. ^*^P < 0.05 when comparing values from age-matched control heart.

To determine if AGE accumulation could alter the expression of SR Ca^2+^ regulatory protein, we performed quantitative immunoblot analysis of SERCA2a and RyR2. SERCA2a pump expression was significantly decreased in the myocardium of untreated diabetic, but not from treated diabetic rats, compared with control groups. We also observed a decrease in RyR2 protein expression in the myocardium of diabetic rats when compared to controls, while the diabetic treated group exhibited similar RyR2 protein levels compared with controls (Figure 6).

## Discussion

The major finding of this study was that long-term treatment with ALT-711, an AGE cross-link breaker, partially restored SR Ca^2+^ handling in cardiac myocytes, by primarily improving Ca^2+^ transient decay compared to the untreated diabetic rats. As a result, ALT-711 therapy partially prevented *in vivo* diastolic dysfunction in the diabetic myocardium of a rodent model of type 1 diabetes.

The STZ diabetic rat model is a well-established model to study insulin-dependent (type 1) diabetes. STZ contains a glucose molecule with a highly reactive nitrosourea side chain, which initiates a specific cytotoxic action on the pancreatic β-cell. A few weeks after STZ injection, rodents develop biochemical and functional myocardial abnormalities, which are the result of chronic hyperglycemia rather than a direct effect of the drug itself. Therefore, diabetic rodents display clinical signs (hyperglycemia, polydipsia, glycosuria, and polyuria) and cardiovascular complications similar to those in human diabetic patients. Since a close relationship between the STZ dose and the severity of diabetes has been demonstrated and since other parameters (such as animal strain, frequency or route of injection, or preparation of STZ, and duration of diabetes) all significantly influence the severity of the model, we previously established in our laboratory a protocol (using a low dose of STZ) to induce a mild form of diabetes and to mimic the early metabolic and cardiac events that occur in diabetic subjects (Lacombe et al., 2007). As a result, the mortality rate was less than 5% and all the rats became diabetic. Importantly, these diabetic rats develop primarily mild diastolic dysfunction followed by mild systolic dysfunction, have prolonged QTs and action potential durations and are prone to arrhythmias (Lacombe et al., 2007, 2010). In addition, this animal model is somewhat relevant to non-insulin-dependent diabetic (or type 2) subjects, who also develop diabetic cardiomyopathy (Boudina and Abel, 2007). Indeed, while initially there is insulin resistance in type 2 diabetes, as the disease progresses there is also insulin deficiency secondary to the exhaustion of pancreatic beta cells (which have produced large amounts of insulin to compensate for the insulin resistance).

Diabetic heart disease, also referred to as diabetic cardiomyopathy, is a major cause of cardiovascular diseases in the United States today. It can lead to heart failure and sudden death, killing ~65% of the patient population (Choi et al., 2002). The presence of LV diastolic dysfunction is an early complication of diabetes and is the first stage in the development of diabetic cardiomyopathy (Fang et al., 2004; Lacombe et al., 2007). Diastolic dysfunction refers to mechanical and functional abnormalities such as impairment of diastolic distensibility, filling, or relaxation of the left ventricle (Aurigemma et al., 2006). The incidence of diastolic dysfunction has been underestimated until the recent advancement of non-invasive imaging tools of cardiac relaxation, such as Doppler flow and tissue Doppler imaging. In particular, the MPI is a Doppler-derived parameter independent of blood pressure and load. MPI increases with worsening of LV diastolic dysfunction, even during the early stages of subclinical diastolic dysfunction (Su et al., 2006). Early determination of this myocardial manifestation of diabetes is of major importance, since subclinical diastolic dysfunction contributes to a four to eightfold increase in risk for congestive heart failure in diabetic patients (Piccini et al., 2004). As previously reported by our group (Lacombe et al., 2007, 2010), this animal model displayed mild diastolic dysfunction, as evident by the alterations of Doppler flow-derived parameters (i.e., increased isovolumic relaxation time and MPI). Since early relaxation is an active process regulated by SR Ca^2+^ handling, impaired myocardial relaxation is characterized by disturbances in calcium homeostasis rather than by fibrosis (Fang et al., 2004; Lacombe et al., 2007). Similarly, we did not detect significant amount of fibrosis in (untreated and treated) diabetic rats, suggesting that the impaired ventricular relaxation rather than increased myocardial stiffness primarily accounts for the negative lusitrope manifested during ventricular filling in our type 1 diabetic model. This is in agreement with previous studies that reported no difference in myocardial collagen (vs. control group) in a similar type 1 diabetic model exhibiting mild diastolic dysfunction (Dent et al., 2001). In addition, it has been suggested that diabetes mellitus can produce diastolic dysfunction before the development of myocardial fibrosis due to formation of AGEs, although the mechanisms were not investigated (Norton et al., 1996; Fang et al., 2004). Therefore, this model of mild diastolic dysfunction allows us to evaluate the effect of AGEs on ventricular relaxation and its underlying alterations in SR Ca^2+^ homeostasis before the development of marked fibrosis.

AGEs are proteins that accumulate in the plasma of diabetic patients as a result of the persistent hyperglycemia and are closely linked with cardiovascular diseases. During diabetes, and to a lesser extent during aging, AGEs also accumulate at an accelerated rate in various cell types (in days to weeks) and produce multiple-organ dysfunction (Cooper, 2004; Hartog et al., 2007). In the heart, AGE accumulation contributes to diastolic dysfunction, by inducing myocardial fibrosis and stiffness (Norton et al., 1996; Asif et al., 2000; Vaitkevicius et al., 2001; Aronson, 2003; Candido et al., 2003; Liu et al., 2003; Bakris et al., 2004; van Heerebeek et al., 2008). However, its role in the development of diastolic dysfunction secondary to impaired ventricular relaxation, determined principally by the rate of resequestration of Ca^2+^ into the SR rather than by an increased myocardial fibrosis, is not known. In the present study, we investigated a novel mechanism by which AGE accumulation functionally impairs SR Ca^2+^ regulatory proteins (especially SERCA pump), by use of an antiglycation therapeutic agent: dimethyl-3-phenacylthiazolium chloride (alagebrium chloride or ALT-711), which chemically breaks AGE cross-links. This compound has been tested in several pre-clinical animal studies, and has been shown to significantly reduce cardiac AGE level in STZ-induced diabetic rats and prevent diabetes-induced structural changes in the myocardium (Asif et al., 2000; Vaitkevicius et al., 2001; Candido et al., 2003; Liu et al., 2003; Vasan et al., 2003; Bakris et al., 2004; Cooper, 2004). In contrast with inhibitors of AGE cross-link (e.g., aminoguanidine), AGE cross linkage breakers prevent but also reverse the cross-link process once it has already been established (Norton et al., 1996). Therefore, one could argue that similar beneficial therapeutic effects of ALT-711, as the ones observed in this study, could be obtained once diabetic cardiomyopathy has been established. Since ALT-711 therapy was administered at the onset of diabetes in our study, further studies will be required to confirm its therapeutic effect in subjects with established diabetes. Overall, our *in vivo* data suggested that long-term treatment with ALT-711 improved the clinical condition of treated diabetic rats, as evident by the increase (although not statistically significant) in body weight. Importantly, ALT-711 therapy partially improved diastolic function, as evident by the attenuation of the prolongation in isovolumic relaxation time and MPI observed in treated diabetic rats. In addition, because of the lack of significant fibrosis in the diabetic myocardium, our data suggested that the beneficial therapeutic effects of ALT-711 were primarily due to improved ventricular relaxation.

In isolated cardiac myocytes of diabetic animals, we observed prolonged Ca^2+^ transient decay, reduced intra-SR Ca^2+^ stores and Ca^2+^ transient amplitude and decreased SERCA2a protein content, which are all consistent with decreased SR Ca^2+^ reuptake during the relaxation phase, as previously reported by our group (Lacombe et al., 2007). Extensive studies have tried to unravel metabolic disturbances and intracellular targets that lead to impaired Ca^2+^ homeostasis and to the diabetic cardiomyopathic phenotype, a complex multifactorial disorder (Poornima et al., 2006). AGE accumulation during diabetes, and to a less extent during aging, could contribute to the observed cardiomyopathy, since AGEs form irreversible cross-links with many proteins with low turnover rates, such as collagen but also intracellular cardiac SR proteins (i.e., SERCA2a pump and RyR2); however, their pathogenic role on excitation-contraction coupling has not been investigated. Since treatment with AGE crosslink breakers has been shown to completely prevent or reduce the formation of AGEs (Wolffenbuttel et al., 1998; Cooper et al., 2000; Vaitkevicius et al., 2001; Candido et al., 2003; Vasan et al., 2003; Forbes et al., 2004), ALT-711 treatment could have a potential beneficial effect on the function of SR Ca^2+^ regulatory proteins. Following ALT-711 therapy, we observed a normalization of the Ca^2+^ transient decay, and a partial restoration of intra-SR Ca^2+^ stores and SERCA2a protein expression in diabetic cardiac myocytes. These data suggested that ALT-711 treatment decreased excess accumulation of AGEs on the SERCA pump by breaking the cross-links that form during diabetic cardiomyopathy, resulting in partial improvement of SERCA activity and SR Ca^2+^ reuptake. The enhanced SR Ca^2+^ reuptake during the relaxation phase of cardiac myocytes resulted in partial improvement of *in vivo* diastolic function in treated diabetic subjects.

In contrast, treatment with the AGE cross-link breaker for 8 weeks did not normalize Ca^2+^ transient amplitude in isolated diabetic myocytes. In correlation with these *in vitro* findings, we observed a moderate, but persistent, reduction of cardiac contractility in diabetic animals treated with ALT-711, as evident by the mild decrease in EF in both untreated and treated diabetic animals. One surprising finding was the persistent decrease in Ca^2+^ transient amplitude in the face of partially restored SR Ca^2+^ load and RyR2 protein expression in the diabetic myocardium after ALT-711 treatment. Since abnormal SR Ca^2+^ release during diastole has been reported in diabetic myocytes (Shao et al., 2007), one could hypothesize that ALT-711 therapy may also improve Ca^2+^ homeostasis by stabilizing RyR mediated SR Ca^2+^ release during the relaxation phase of diabetic cardiac myocytes and that AGE accumulation may also impair RyR function leading to diastolic SR Ca^2+^ leak. Therefore, these data further support the concept that AGE accumulation may play a larger pathogenic role during diastolic dysfunction and diastolic heart failure than during systolic dysfunction (Hartog et al., 2007).

## Conclusions and clinical significance

Treatment with an AGE cross-link breaker partially attenuated the alterations associated with cardiac function and SR Ca^2+^ handling during diabetic cardiomyopathy. Since diabetic cardiomyopathy is a multifactorial disorder, these data suggest that AGE accumulation contributes to the impairment in excitation-contraction coupling by altering the function of SR Ca^2+^ regulatory proteins, leading to a decreased ability for the diabetic myocardium to relax. Therefore, findings from this study provide novel mechanistic insights related to the pathogenic role of AGE accumulation on SR Ca^2+^ handling in cardiac myocytes. Finally, since there is currently a lack of specific therapy to improve LV relaxation, findings from this study could have direct practical implications for the development of therapeutic strategies for patients with diabetic cardiomyopathy.

### Conflict of interest statement

The authors declare that the research was conducted in the absence of any commercial or financial relationships that could be construed as a potential conflict of interest.
